# Hospital Meetings

**Published:** 1901-03-30

**Authors:** 


### The Institutional Workshop.

### HOSPITAL MEETINGS.

### NATIONAL HOSPITAL FOR THE PARALYSED AND EPILEPTIC.

The Medical Staff and the Board.

*Appointment of a Committee of Enquiry Unanimously Agreed to.*

*Governors Selected to make the Necessary Arrangements.*

A special meeting of Governors of the National Hospital for the Paralysed and Epileptic was held at the Hospital, Queen's Square, Bloomsbury, on Saturday, March 23rd, for the purpose of considering the following resolutions:—

(1) "That inasmuch as the demand of the medical staff for direct elective representation on the Board of Management has been expressly based upon certain allegations contained in a statement that was issued by the medical staff to the Governors in June, 1900, and inasmuch as it would be impossible to test the accuracy of such allegations at a meeting of the Governors, it is hereby resolved that an independent inquiry into the accuracy of these allegations ought to precede any reconsideration of the demand of the medical staff."

(2) "That it is essential (*a*) That the inquiry mentioned in the preceding resolution should be presided over by a distinguished member of the legal profession. (*b*) That the method of conducting such enquiry should be determined by the persons appointed to hold the enquiry. (*c*) That if the question of medical representation is included in the enquiry, the enquiry should not be limited to the exact form of medical representation demanded by the staff."

The Right Hon. the Earl of Halsbury (a member of the Board of Management) was voted to the chair.

The Chairman said the first item of business on the agenda was one which would certainly not cause any difference of opinion: it was to move that an address of condolence be presented to His Majesty the King (Hear, hear.)

The motion was agreed to.

The Chairman: The next business is that which has practically caused the summoning of this meeting. I am happy to believe that the situation, which undoubtedly is most injurious to the hospital, seems to me, by reason of various conversations with different members both of the medical staff and the Board, to be very nearly approximating to a settlement, and I certainly, in consenting to take the chair at this meeting, wish to profess myself as absolutely impartial—(hear, hear)—but at the same time I do not wish to dissociate myself from my colleagues on the Board, and I will explain to you how I think those two propositions are perfectly reconcilable. I am very strongly of opinion that the question which, I am afraid, has given rise to the difference amongst us, is one which may very fairly and properly be a subject for debate, although not, I think, to cause anything in the nature of acrimonious discussion amongst us. After all, we must all remember—I am sure I strive to do so—that we are engaged in the support of an object which we all have at heart—(hear, hear)—namely, the relief of suffering; and speaking not simply as a member of the Board of Management but as one member of the State, nothing would seem to me to be more injurious to such an institution as this than a sort of feeling that there was a violent controversy between those who have the responsibilities and those whose great eminence, skill, self-sacrifice and devotion to duty—(hear, hear)—have undoubtedly been of inestimable benefit to those who are the objects of this institution. But I decline to look at this meeting as if it were an array of two hostile forces. It is rather, I think—and I hope it will be conducted in that spirit—a meeting of us all who are desirous of doing what is best for the hospital and for those who are the patients receiving its benefits; and if we are all actuated by that desire, and we all remember that we are not always absolutely in the right—because if we differ we cannot all be absolutely in the right—and make some allowance for the prejudices and feelings of others as well as for those of ourselves, I am quite sure that at such a meeting as this we shall arrive at a final conclusion in this matter, which no one can doubt is most injurious to the interests of the hospital. That is all I have to say upon the matter which engages our attention to-day. For my own part I should regard the question which, as I say, gave rise originally to the question now before us as one upon which really my mind is still open, although I did vote with the board—I mean the question of the representation of the medical staff on the board. That is not the question before us to-day; but there is no doubt that some of the statements which have been made—I will not call them charges, and I will not say on whose behalf they were made—are undoubtedly statements of fact, which, if they were true—and when one is speaking of what is to be a matter of investigation one cannot say whether they are true or not, and I do not wish to prejudice the question in the least—but if they were true and were not remedied I personally would not consent to be a member of the Board of Management for another day. I am quite sure we will all feel that these are matters which ought to be decided, and of course it would be perfectly idle to suppose that at any meeting of this sort we should submit allegations or denials with the least prospect of determining the accuracy of such allegations or denials. I understand this meeting is practically called together to decide in what manner and to what extent these questions and facts should be investigated, and so far as I am concerned, as soon as this question is decided, it will be absolutely for the governors to consider and determine—and I will be the first to desire to bring it forward—the other question, namely, the representation of the medical staff on the board. That is all I have to say, and I hope, under the circumstances, that we may be able at no great length, and certainly in no inharmonious spirit, to determine the question which is now before us for decision. (Hear, hear.)

Mr. George W. E. Russell, in the course of a lengthy statement, said, I have been asked by my colleagues on the Board to move the first of the two resolutions which are before you. I may begin by saying that it is really to me a matter of sincere regret that I have been forced by circumstances again into a position of prominence in connection with this rather unhappy dispute. It is certainly by no choice of my own that I figure on this occasion as the mover of the first resolution. I am here merely in compliance with the request of my colleagues, which I did not feel at liberty to disregard, the more so as owing to the unfortunate circumstance of illness, the number of our Board who are able to take an active part in the proceedings at this moment has been sensibly reduced, and of course any services I was able to render to my colleagues under these circumstances they had an absolute right to demand. The Lord Chancellor has stated with, of course, perfect accuracy, that this resolution which I have to deal with concerns not the rights and wrongs of what I may call the constitutional question—the question whether the medical staff should obtain direct elective representation on the Board—but a separate, though not unconnected proposition. We are asked in the terms of this resolution to affirm that an independent enquiry into the allegations made in the statement addressed to the governors last summer should precede the enquiry into what I term the constitutional question. I am not going, therefore, to re-argue the merits of the question of direct elective representation. Last summer it became my duty to state my views on that subject, and there is no reason, even if it were relevant and proper, to repeat them now. But I should like to recapitulate—I will not say for the information of my hearers, because everybody has had abundant means of deriving information from the literature which has been circulated, but rather of refreshing the memories of my hearers—the steps which brought us up to the present position. The demand of the medical staff is an old demand. It was formerly made, I think, at least ten years ago. It was opposed by the Board of that date, and fell for a time into abeyance. Other methods of communication between the medical staff and the Board were proposed in preference, and were more or less steadily acted upon. One of these methods was that the medical staff should send two delegates to meet the Board to discuss medical questions when such arose. That is a form of procedure which was in use until a very recent date. But in February, 1899, a rather acute controversy arose between the medical staff and the Board. The question then at issue was not, as I conceive, a medical question. Throughout our previous proceedings—I think I am right in saying—the Board had always been not only willing but most eagerly anxious to obtain the assistance and even the final judgment of the medical staff on all matters relating to science, medicine, and the care and treatment of the patients. That clearly was a matter with which we on the Board of Management were not competent to deal. But the question raised in the spring of 1899 was a question of a very different sort. Notoriously the expenses of this hospital in working and management have increased very rapidly. It would carry me altogether out of my province if I were to attempt here to trace the causes of that increase, but the fact is apparent to anyone who examines our accounts. It became, therefore, necessary to look round and see if we could not by some legitimate means raise the income of the hospital in something like the proportion of the ever-increasing demands upon it, and it occurred to Mr. Rawlings, our indefatigable secretary—I speak subject to his correction, but I think I am right in saying this—who, whatever else he may be, has been a wonderful Chancellor of the Exchequer to the hospital—I think it occurred to him in the first instance, and he represented to the Board that a good way of raising money and increasing our income was to sanction, though not to enforce, a small payment by such out-patients as were ready and willing to make it for the medicine supplied to them in this place. This payment was not to be made for treatment. All that was suggested was that as they received their medicine here they should be invited and encouraged, though not forced, to give for it the very trifling sum which it cost the hospital. That seemed to us on the Board not to be in any sense a medical question. It seemed to us to be merely a question of finance and administration, and those are the questions which, if any, we consider ourselves competent to deal with and bound by our office to deal with Therefore we proceeded to sanction this alteration in our working system. The result, I am told, is that we are making some £800 a year for the hospital, which cannot be regarded as at all an inconsiderable contribution towards our income. It is a clear gain to that extent. No hardship is inflicted on anyone, and no one is enforced to pay, and I am informed by those who see the patients that the better sort of patients—those who are a little better off than the others—are ready and eager to make this small acknowledgment of the benefit they receive in the hospital by paying for the medicine supplied to them here. At any rate, that was decided upon, and I am bound to say it was decided upon by the Board in opposition to what we knew to be the sense of our medical staff. At that point, or shortly afterwards, there arose a regrettable misunderstanding, to which reference was made last August, and there can be no harm in referring to it again. This alteration in our practice was the subject of discussion and controversy between the medical staff and the Board—the medical staff being at that time represented by two delegates sent to discuss the matter in the Board-room. There is no doubt at all that our intention was that these two delegates should be present throughout any discussion on the subject which occurred, and it was owing to an accident—I can call it nothing else, for it was in no way the design of anyone on the Board—that at the adjournment of the discussion the two medical delegates were not invited to be present; so that the very last step was taken in the absence of the two representatives of the medical staff. That was a matter to us of sincere regret and that regret has been expressed so fully and frankly on previous occasions that I am almost doing a work of supererogation in referring to it now. But the untoward part of it was that the medical staff then declined to continue the system by which they were represented, in conjunction with the Board, by two delegates. They said, in effect, that it had broken down through our fault and they declined to be parties to a continuance of it, thereby producing a deadlock in the official or formal communications between the medical staff as a corporate whole and the Board. I draw that distinction because communications went on freely between the individual members of the medical staff and the Board and the secretary-director, and the work went on as before. I am not aware that any practical hindrance arose. But there was this regrettable incident, that the medical staff declined any longer to adhere to the plan which had seemed to us to work reasonably well—for the delegates to attend when matters came before the Board that were strictly of medical interest and importance. The next step in the controversy, if I may so call it, came upon us as a very great surprise. A document dated, if I remember rightly, May, 1900, but circulated only on or about the 10th of the following month, was addressed and sent out to the Governors. Most of us are familiar with it. It commenced thus: "We, the members of the medical staff, think it our duty to bring before your notice the fact, that to our knowledge the administration of the hospital is very unsatisfactory, and that the reputation of the institution is in danger of being seriously compromised." The conclusion is: "In closing this brief survey of the present condition of the hospital, which the medical staff feel it their duty to submit to the Governors, they call attention to the following conclusions, which have been unanimously agreed to: 1. That to safeguard the interests of the patients and the reputation of the National Hospital by co-operation of the Board of Management and the medical staff, it is indispensable that the medical staff be directly represented on the Board of Management. 2. That in view of the impossibility of their continuing the work at the National Hospital under existing conditions, the medical staff appeal to the Governors to effect this necessary change in the management of the hospital." That document was circulated to the Governors of the hospital without any reference to the Board as such. Of course those who were members of the Board and also Governors would receive it in the ordinary course; but it did not come before us officially and as a body. I confess the rest of it filled us with surprise. Coming to the allegations we read: "The first matters on which the medical staff have to report to the Governors are those affecting the immediate interests of the patients, as follows:—(1) The diet of the patients. (2) The condition, care, and treatment of the patients. (3) The nursing of the hospital. The other matters relate to the constitution of the hospital and its system of management, namely: (4) On the cause of the maladministration of the hospital. (5) Details of the manner in which the present administration has prevented the co-operation of the medical staff and board of management." The two items (4) and (5) may be disregarded. The items I speak of as being the allegations that caused us such astonishment were the statement, in very uncompromising terms, that the diet was wrong in quantity and quality; that the condition, care, and treatment of the patients was wrong; and that the nursing was not properly and efficiently organised. If those allegations are borne out by facts, as his Lordship in the chair has said, each one of use would feel that a very heavy responsibility lay upon him, and it was therefore a very great source of astonishment and regret to us to find that our medical officers had thought it necessary to commit themselves to the statement of so very grave a charge—statements which I must remind my hearers amount to a very heavy indictment against the persons charged with the immediate oversight of the hospital, and under that term I include, of course, not only the Board, but our very efficient representative, Mr. Burford Rawlings. The point is that none of us knew that any of these mischiefs were alleged by anybody. As far as I was concerned they came upon me as absolute novelties, and, although not fond of talking about oneself in public, in one sense I am justified in quoting my own experience, because ever since I have had the honour of being connected with the Hospital—which originally I joined at the urgent request of an honoured member of the medical staff, Sir William Gowers—I have made it my invariable, constant habit to be in the wards as much as possible, and to see as much as I could of the actual state and comfort of the patients. My ears have thus been open to any complaints that might be made, my eyes have been open to any abuses that might be visible, and for my own part I believed everything was working as well as it could be in the hospital; that the patients were well looked after, were thankful for the benefits received, and that a general tone of contentment and cheerfulness reigned supreme. Great was my astonishment and that of my colleagues when we found that our administration was really so faulty as was alleged in the statement circulated to the Governors. Mr. Rawlings was equally unaware that there were any serious matters of complaint. We were, in a word, taken by surprise, and we might have thought it would have been better if our medical friends had made these representations direct to us, as the executive authority of the hospital, in the first instance and then, if they were not satisfied, they could be reported to each of the Governors. But that was not the course which recommended itself to them, and they were of course within their rights in going to the Governors in the first instance. But then we were also within our rights in investigating the allegations of mismanagement on our own account and on our own authority; because, after all, supposing the facts were as alleged, the guilt—I can call it no less—would lie on those who are directly employed and paid; the nurses, servants and dependents of the hospital, and upon ourselves for not having exercised the necessary vigilance in looking after the patients. We formed a subcommittee, which sat at great expense of time and comfort and convenience during the hottest weather of last summer and investigated high and low—in wards and kitchens, and in every corner and section of the hospital. We examined all the witnesses who were the most likely people to be able to contribute anything to our knowledge—the lady superintendent, the ward sisters, the cook, and the nurses and servants of all descriptions. We reported to the Governors that the charges had what I should call no substantial basis or foundation and could only be justified or made good in such small and irregular instances as must, I imagine, always occur in the food arrangements of a very large institution which is fed from one kitchen. When we had made up our minds on the subject and reported to the Governors, it was judged to be necessary to summon a meeting of the Governors, and that was done in August. Of course it was an inconvenient time: it was as inconvenient to us as to anybody else. No one would choose August as a suitable month for a public meeting in London. But it was the earliest possible date after the allegations had been made and inquired into, and we felt we ought to allow no time to elapse before we put the results of our enquiry before those who, after all, are our governors and judges. On that occasion the constitutional question was argued, and was decided against the claim of the medical staff. We all remember that the moment that decision was made known a rather fierce and acrimonious controversy began in the public newspapers. We were then in what is known as the "silly season" when the newspapers are only too thankful to have a little extra pabulum, and this really rather small dispute was, by journalistic enterprise, magnified and distorted to the largest possible dimensions. Contributions poured in from some correspondents of whom I will only say, with great respect, that they were not absolutely well informed of the facts at issue. The only part that I myself took in that controversy was my speech in the chair in this room at the August meeting, and with regard to that I should like to be allowed to make one slight personal explanation. Speaking from the material supplied to me by our professional advisers, I stated, in quoting the practice of other hospitals in London, that Brompton Hospital for Consumption was one of those which did not admit of direct medical representation on its Board of Management. As soon as I had stated that, a letter appeared in the *Times* from a very highly respected member of the profession, Dr. Pollock, stating that I was so far misinformed that at the hospital all the medical staff were honorary members of what I think is called the committee of management. Recognising that authoritative contradiction, I made haste to express regret that I had been misinformed and had repeated the statement. It happened the other day, however, that I received from Dr. Pollock himself, in reply to a question I put to him, a rather interesting piece of information, which was to the effect that, although at Brompton the members of the medical staff are *ex officio* members of the committee of management, they are only entitled to appear there and speak but are not entitled to vote. That is so extremely different a position from that which is now claimed on behalf of our medical friends here that I feel that, after all, my original statement, which I made on the authority of our officers here, was not, substantially, very wide of the mark. (Hear, hear.) I am reminded that Dr. Pollock also said that in practice the medical men at Brompton only attended the committee of management when medical matters came up for discussion. During the autumn months this controversy raged with some acrimony, and by degrees we learned that many of those who had the best right to be concerned in the matter were not at all satisfied with the result of either our domestic enquiry or the vote taken at the August meeting. We had hoped that that enquiry and that meeting would have brought the controversy to an end; but when we saw there was uneasiness in the minds of many of those who have the hospital sincerely and seriously at heart, we came to the conclusion that a fresh enquiry into the allegations of mismanagement should be undertaken by some competent exterior authority. I need hardly assure my hearers, among whom I recognise some old friends, that there was no inclination on our part to shrink from the fullest investigation of what we had done—imperfectly, perhaps, but with loyal goodwill—in the administration of the hospital during so many years, and we welcomed the prospect of an enquiry conducted by some one accustomed by the nature of his occupation and the habits of his life to sift and weigh evidence, and even conflicting evidence, if need be. We were not at that time prepared to submit to that enquiry any question except the allegations of mismanagement. We were not disposed to submit the constitutional question of the representation of the medical staff on the Board of Management. It became evident, however, from communications we had with our medical friends and with others, that at any rate a very much greater degree of public satisfaction would be attained if we waived our own previous opinion and allowed the constitutional question of direct representation to be included in the scope of enquiry; and with a view to carrying out that two-fold enquiry, we invited, through the intervention of the Lord Chancellor, the good offices of an ex-judge of the Supreme Court, Sir Ford North. Now, although he was, of course, highly competent to undertake and carry through successfully the duty which we proposed to entrust to him, and although he was willing to place his services at our disposal, the medical staff, speaking through the medical committee, declined to recognise an enquiry conducted by Sir Ford North, appointed by us, and refused to render that assistance in the way of appearing before the committee and giving evidence which would, of course, be necessary in order to secure the thorough investigation of the charges of mismanagement which, to our thinking, are of such vital importance to the interests of the hospital. I believe it was in December last that we consented to the inclusion of the constitutional question in the enquiry, but when we consented to that we stipulated that whoever should hold the enquiry should be entitled to consider not merely the demand of the medical staff from the particular form of representation that they desired, namely, direct elective representation of two members on the Board, but any other form of representation such as might be suggested by the experience of other hospitals, or might commend itself to the minds of any of us as a more workable arrangement. Almost at the eleventh hour, hoping still to stave off a pitched battle—if that is not too strong a phrase to use—we suggested to the medical staff to consider whether any of these alternative forms, for some of which our esteemed colleague, Mr. Pearman, was responsible, would be acceptable to the medical staff; but we were told emphatically and plainly that they would be satisfied with nothing but the one form on which they had already set their hearts, the direct election of two representatives to sit on the Board. (Hear, hear, and cries of "Time.") The cries of "Time" are very apposite, because I have just come to the end of what I have to say. The history of the case has now been adequately presented, and we have arrived at a point where I hope that a decision salutary to the interests of the hospital may be arrived at with something like practical unanimity. We desire an enquiry not only into the allegations of mismanagement of our patients — allegations which are still unproved and unwithdrawn — but also into the question of some mode of securing medical representation on the Board. (A voice: "It is not in the motion.") The second resolution covers that. The first resolution which we are asking your judgment on, is only a question of precedence. We ask you to affirm by the first resolution that the enquiry into the question of allegations should precede the constitutional question of the representation of the medical staff on the Board. The demand for representation was based on allegations in the terms I read you from the circular letter. (Cries of "No.") The speaker re-read the portion of the document affecting the point (par. I. of the "Conclusions") then proceeded: That surely bases the claim to representation on the alleged insufficiency of the existing system. (Hear, hear.) On the very first page of the circular it says: "We regret that in consequence of the difficulties we have experienced of obtaining from the Board of Management real appreciation or permanent redress of the evils now prejudicing the Institution, and primarily due to its present constitution, we are compelled to cease communication with the Board and to address you directly concerning them. We do so in view of our common interest, namely, the welfare of the patients of the National Hospital." We therefore only ask now that you should instruct whoever is charged with the conduct of this enquiry to investigate the allegations of mismanagement before proceeding to enquire into the constitutional question. If the allegations of mismanagement turn out to be well-founded, no doubt they will be a matter for the very serious consideration of all the Governors of the hospital. If, however, they have no existence except in imagination and in the heated language of this controversy, then it will be for the Governors to consider whether the gentlemen responsible for making allegations so severe and ill-founded can with advantage to the hospital serve on its administrative body. (Applause).

Mr. F. C. Capel seconded the resolution, remarking that in doing so he was taking the place of the treasurer, Lord Harrowby, who was unable to be present.

Mr. Melville Green, who was received with applause, rose to move the following amendment: "That a full and independent enquiry be at once instituted by the Governors into the statement issued by the medical staff in May, 1900; the facility of intercommunication between the medical staff and the Board; the demand of the former for direct representation on the Board; the position, functions and acts of the secretary-director; and the constitution, rules and management of the hospital generally." He said: The motion before the meeting alleges that a demand made for the past 10 years or more is based upon a paper issued last spring. That is a difficult statement to assent to, to begin with. As to the next sentence, that it would be impossible to test the accuracy of the allegations at a meeting of the Governors, it is, of course, impossible to ascertain a great many things in a public meeting like this, and therefore there should be an enquiry, full and complete, and, to use the secretary's word, exhaustive. Then we are asked to resolve "that an independent enquiry into the accuracy of these allegations ought to precede any reconsideration of the demand of the medical staff." It is a very remarkable resolution. It does not say there is presently to be an enquiry into the demand of the medical staff; it does not even say there is to be an enquiry into the accuracy of the allegations. It affirms only that the enquiry should be two, and not one, and that one of those enquiries should precede the other. That is the whole effect of the resolution, and I am going to ask you to dissent from it altogether. I do not want to refer to history, because I am of opinion, as you my lord are, that the object of us all should be to bring about harmony as soon as we can, and references to history, if they are avoidable, are only likely to promote further irritation. But I must go back as far as August 11th, and I may say here I was very pleased with two of the remarks that fell from the mover of the resolution, one of which was that the resolution arrived at in this room on August 11th was arrived at in the "silly season." I quite agree. (Laughter.) On August 11th, and up to December 10th, the attitude of the Board was unmistakably immovable. The strongest language possible was used by Mr. Russell on August 11th as to the absolute impossibility of having any enquiry into this question of the demands of the medical staff. He refused it as strongly as possible, and mine was the only voice—I suppose everybody was out of town, but I came up to town—that demanded a full and complete enquiry. (A voice: "We did not know of the meeting.") I daresay you did not, and I am going to repeat therefore, to-day, exactly what I said then. It is necessary, after a speech of more than half an hour from Mr. Russell, to call attention to the matter as it strikes me in supporting the amendment that I have the honour to move. Up to December 10th, the Board were anxious to refer—exactly as they are to-day—the domestic management of the hospital by itself, to someone to report upon; for it must be borne in mind, after all, that this reference can be nothing more than to report. The actual carrying out and giving sanction to the recommendations of that report must rest to us here as Governors. No one else can do anything. (Hear, hear.) On December 10th, the Board—not spontaneously, not of their own idea, but because their counsel showed to them what a false position they were taking up—began to allow of a wider enquiry. I think it took a few days—it came by degrees, if I remember rightly—and they added that as a concession (though I think the staff was very unwise to regard it as a concession) the persons to make the enquiry were to be three instead of one. I do not, for my own part, attach any importance to a tribunal consisting of three people, one named on each side and a third between them. It is the common way of having two arbitrators and an umpire. I have seen a great many such enquiries in my time, being a solicitor, and I do not ever remember the umpire being in a minority of one. Whatever view he takes one or other of the first two will take, according as it supports the wishes of the party who nominated him or those of the opposite party. That is human nature. A tribunal of three so nominated is no better than a tribunal of one; or, at least, no better than this, that it secures his listening with great attention to his two colleagues—that is all. Therefore, I do not think there was much gained by that. Those negotiations have broken off, and it is immaterial, I think, to say how or why that is the case. If I were asked to say that either side had acted with uniform wisdom I could not. I have had the advantage of seeing the formal resolutions that have passed between them, and I should not be able to say so. Now, so long as there was a chance of an enquiry on those lines the Board were willing for a large enquiry, but directly that chance is over we get the motion that is put forward to-day. Coming back to the resolution, Mr. Russell has told us it includes an enquiry into the whole subject. You may judge of that. Does it? Read it. It does nothing of the kind. We are told that the staff and the Board are not in touch with one another, and that, in consequence of their not being in touch, mischiefs ensue in the hospital. Well, I have known—as I daresay many here have known—a firm of two or three members who were not on speaking terms with each other, all the communications being done through the heads of departments. It does not need any knowledge of the world to be quite sure that the business of that firm is not carried on as well as if the partners were in touch with each other. (Hear hear.) We have had it proved up to the hilt that the Board is not in touch with the staff. Mr. Russell's own words, in August, told us that the secretary-director —that anomalous officer—was an excellent medium of communication between the staff and the Board. (Laughter.) Well, I do not think I need labour the point, or that it is necessary, and indeed essential, that the staff and the Board should be in touch. (Hear, hear.) There may be many means by which this could be accomplished. Sir Henry Burdett told us there were several. But every mode that has been tried has failed. We are told that there are hospitals that get on very well without the representation of the staff on the Board; but we have not been told of one single hospital where there is representation—and there are a great many—of the staff upon the Board that produces any evil result. (Hear, hear.) Why are not the hospitals quoted where the mischief is done? The gentlemen on the Board, and Mr. Rawlings, have every opportunity of communicating, but they have not told us of a single instance of that kind. I do not know how the Governors are to settle that. We cannot enquire of all the hospitals, but a committee of enquiry could, and could obtain the information. We can only judge for ourselves upon our own experience which in my case is very limited. I served for some years on an infirmary committee—my town does not boast of an hospital—and there we had a rule by which not only every member of the medical staff was a member of the committee, but there was a rule that the senior member should take care that when he could not attend himself one of his colleagues should, so that we never had a meeting without one of the staff being present. A friend and neighbour of mine wrote me, quite spontaneously yesterday, thus: "If the experience of one who has for nine years been chairman of a hospital committee of which the whole of the honorary medical staff are *ex officio* members be of any service to your fellow-governors, you are quite at liberty to make use of it. In my opinion some attendance of the honorary medical staff is necessary, and I should immediately resign the chairmanship of my committee if I was deprived of such assistance." I do not put that forward as being of the slightest authority. We Governors must judge of the matter out of our own limited experience, or else have an enquiry about it, and the Board, by their resolution, will not give it. It is nonsense to tell us they mean something more than is in it. I have most confidence in resolutions which mean more than they say. If they mean it why do they not accept the amendment? That amendment provides for all kinds of representation to be considered, and is surely wide enough. As to "the position, functions, and acts of the secretary-director," that is more necessary than anything else. The position of a secretary-director is unknown elsewhere in the hospitals of this country, and we Governors from all parts of England do not quite know what it means. We only see that it is a very dangerous thing. As to the last clause of the amendment, you will hardly believe, if you have not gone through the rules as I have, that they are so framed that we, the Governors, are hampered on every hand from governing this hospital. ("Hear, hear.") We have not heard any reason why an enquiry should not comprehend everything that I propose, nor have we heard any reason why the enquiry should be cut into two parts. If the state of the case be, as I say, that the whole thing is one question, why cut it into two parts? It is suggested that the staff would be satisfied if they were put on the Board without any enquiry into these other things. I do not know whether they would or not. ("No.") Well, so much the better. But I know this, that those things would never have arisen if the staff had been on the Board. The Board do not seem to understand the sort of influence that two, or even one, man exercises on a Board. It is not like an outside body that comes and meets them. An outside body that comes and meets them comes to make some large proposal or some complaint, and is dealt with in that sort of way. But a man who is a member of a body is often there: he knows his colleagues; their minds are constantly in touch, and on all sorts of subjects they gather from him. The Board themselves, being human, would be instructed by the presence of two doctors, just as the two doctors who might sit with them would be instructed in business matters. The presence of members of different sorts on a committee has that effect, and it is this that the

Board are not only anxious not to have, but they refuse to enquire whether it is a good thing and how it works in other hospitals. I think you will agree with me that the whole subject ought to be enquired into. (Hear, hear.) The Board, throughout the whole of the papers they have sent us, have spoken of the influence of two members of the staff as the "domination." They speak as if the presence of two doctors on the Board would mean the domination of the medical element over the lay element. The policy of fear which characterises the Board is a marvel to me. They are afraid of this full enquiry. ("No.") Yes, you are. You do not move it. ("Yes we do.") They are afraid of being dominated over by two men—a dozen of them! But you see they say "they are such men!" (Laughter.) So they are. They are accustomed to dominate in the sick chamber. But I would point out that the medical staff themselves meet. I do not know how they manage. Do two of them dominate over the rest? Because none of them can do anything but dominate, and therefore it must be an impossible thing to have a meeting of the medical staff. Doctors differ, but I do not know that doctors dominate more than other people. The charge against the Board is that they are dominated over by one man. Will you believe that the inner consciousness that they must yield to two is proof that they do not yield to one? I cannot. Now we come to the proposal that we should institute an enquiry. The manner of instituting it is left by my amendment, as by Mr. Russell's resolution, for subsequent resolutions to carry into effect. If you carry my amendment you affirm that there shall be an enquiry in as wide terms as it is possible to frame; or if they can be made wider by any member of the Board I am only too anxious to adopt such additional width. I would point out that the whole conception of my committee and that of the Board's committee is quite different. They want a sort of tribunal that will deal with the matter as if it were a private lawsuit between the staff and the Board and give a verdict either for the plaintiff or the defendant. I do not. I want some people who will advise us. The responsibility rests, not with the staff or the Board, but with us. (Hear, hear.) The circumstance that we need not come forward or do anything at all does not in the least diminish our responsibility when such a question as this is raised. We are bound, from the accident that we fill the position of Governors, to accept the responsibility. We cannot discharge that responsibility well unless we have a full and perfect enquiry and a complete report, and it is to help us in that way that I want an enquiry, and not to decide a lawsuit or go into matters in any such spirit; but to see what ought to be done to benefit this institution. (Applause.)

Sir James Crichton-Browne, F.R.S. (one of the vice-presidents), in seconding the amendment, said the allegations of mismanagement seemed to have become a matter of secondary importance. What the Governors had now to decide was the very existence of this National Hospital, which, thanks to the labours of the medical staff, had obtained world-wide renown. Attention should not be diverted from the important question of the representation of the medical staff on the Board. In the Board's resolution it was alleged that the demand of the medical staff was expressly based on certain allegations which were to be submitted to enquiry. That statement was entirely—he was afraid he must say deliberately—erroneous, because it must have been known to whoever prepared the resolution that the demand for representation had been persistently urged by the medical staff for ten years past, and that the allegations were brought forward not as the initiation of that claim, but as an illustration of the fruits—the inevitable fruits—of the present mode of management. (Hear, hear.) The small, trivial, petty annoyances, the little indignities, the great obstructions with which the medical staff, he understood, had had to contend for a long series of years, were culminating, as they had all along promised to culminate, in evils injurious to the profession; and what they as Governors had to do was to go to the root of the matter and effect a reform which would at once put an end to the evils and the causes and sources from which they had proceeded, and to see that any enquiry instituted should be thorough, searching, and conclusive. If they authorised the preliminary enquiry which was suggested by the resolution they would not, when that was finished, be any further than they were to-day. They would have wasted time and the funds of the hospital, and the burning question of the representation of the medical staff on the Board would still remain to be decided; and remembering as he did that the Board of Management had said in print more than once that that representation should have their unyielding opposition, he could not but conclude that the enquiry that was proposed was somewhat evasive and illusory, and was only calculated to cause delay or perhaps to get rid of the demand of the medical staff by a side issue. He had had the curiosity to examine for himself the statement of the medical staff to June, 1900, to which allusion had been made, and what he could not understand was why that statement was not at once submitted to the judgment of the Governors to whom it was addressed. The Board of Management, without any reference to the Governors, dealt with it themselves, and appointed three of their own number to enquire, not into the whole indictment brought against them, but into three selected counts of the indictment; and after that enquiry, at which the accusers—the medical staff—were not represented, and of which no minutes of evidence had been published, they called the Governors together at an irregularly summoned meeting in the height of the holiday season, to ratify their own acquittal of themselves. What the Governors had now to do was to see there should not be any repetition of proceedings of that sort. If there were to be an enquiry it must be thorough, searching, and complete, and it must be impartially conducted. It must deal with all the allegations that had been made by the medical staff. The statement of that staff must be the subject-matter of the enquiry whenever it should be held. He was much mistaken if the governors would consent to any limited enquiry. They would, he thought, insist that all allegations having reference to the constitution and administration of the hospital should be included. The allegations of the doctors was not limited to questions of diet or nursing or the condition of the patients, but included complaints as to the anomalous position and powers of the secretary-director; as to the abuses of the out-patient department; as to the exaction of money from patients attending this charity; as to deficient accommodation for the nurses; as to the position of members of the medical staff who subscribed as Governors but by reason of the fact that they give their services gratuitously to the hospital were deprived of their votes as Governors; as to the rules of the hospital, and various other matters. The medical staff did not shrink from an enquiry—they courted it. They were prepared to establish every statement they had made, but they declined to have their professional status in the hospital postponed to a preliminary enquiry as to the drawsheets and the boiling of potatoes. They said plainly, and, he thought, wisely—"We cannot any longer acquiesce in the present state of things. In the interests of this hospital, of which we are so proud, in the interests of the patients to whom it is our duty and privilege to minister, in the interests of our own self-respect, we demand a full and searching enquiry. We require access to the Board of Managers. We have asked for a very modest representation on the Board—two out of twelve

—and we feel sure if that be granted, all need for enquiry wil disappear, all dissensions will cease. But we challenge an immediate decision. For years past we have been pressing our plan on the Board of Management. We have tried all sorts of substitutes which they have suggested, and they have ended in failure, and now we challenge an immediate decision. The matter is in the hands of the Governors, and by their judgment, fairly and honestly as certified, we shall abide. If they accept our proposal we shall, as heretofore, zealously exert ourselves for the welfare of the hospital. If they reject it and declare that the affairs of the hospital are to be placed unreservedly into the hands of the secretary-director, we shall accept their verdict and retire from the field." For some time past it had been seen what must be the inevitable consequence of the line of conduct which had been pursued by the Board, and in looking over the correspondence which had taken place he (the speaker) was impressed with the belief that the secretary-director had been quietly working for the resignation of the medical staff. ("Oh!") At any rate, in that exegetical, tortuous, curious document, with its copious rubric, which had been circulated lately, he contemplated the resignation of the medical staff with great complacency. "The hospital," said the secretary-director, "may encounter worse evils than the resignation of its medical staff." He differed from Mr. Rawlings. (Hear, hear.) The resignation of the medical staff must mean the irretrievable ruin of the institution. (Hear, hear.) They had on their staff eighteen of the most eminent physicians and surgeons in London—men whose names were known and respected wherever medical science went, and whose special skill was of incalculable benefit to the suffering; and surely it was a significant fact—remembering what Mr. Russell and Mr. Melville Green had said, that doctors proverbially differed—that these eighteen physicians and surgeons were unanimous and solid over this question. If they lost these gentlemen how were they going to replace them? Mr. Burford Rawlings calculated that there were blacklegs in all professions. Let him (the speaker) tell that gentleman that on this question the medical profession was unanimous. Their leaders and journals had spoken out with no uncertain voice, and if the staff of this hospital resigned there was no medical men who would consent to the humiliation—the ignominy, Sir William Broadbent had called it—of stepping into their shoes; at least, there were no medical men who would do so whom it would be prudent to entrust with the life of a sick cat. The hospital was a medical institution, and every detail of its management must be in accordance with medical consideration, and it would be just as reasonable to deprive the Admiralty of its sea-lords and leave our Navy entirely in the hands of civilians as it was to leave a hospital board of management without medical guidance and advice. Where the medical staff was paid—as in the old London hospitals—one method was adopted; where honorary officers were working there was another method in vogue; but in all of them medical assistance and advice was obtained in the same way save in this National Hospital in Queen Square, in which it appeared to be tabooed. And yet it was there that medical assistance and advice was especially necessary, considering the obscure nature of the diseases treated and the peculiar nature of the treatment demanded. Mr. Burford Rawlings had insisted that the exclusion of the medical element from the board of management was necessary in order to prevent the hospital from being prostituted by experimental practices and converted into a scientific laboratory or workshop. He was not surprised at the scorn and indignation with which the medical staff had received that shameful appeal to the governors for their votes. Every hospital and sick room in this country was a scene of experimental practices, for the administration of every dose of medicine was an experiment; and it was fervently to be hoped that the medical staff of this hospital, so long as theer was one, would continue to hold in view one of the great objects of the charity as set forth in its charter on the very first page, viz., that they would continue to investigate and study diseases of the nervous system, and to increase our knowledge of them to the inestimable benefit of humanity. (Cries of "Time.") To suggest that the medical staff desired to have representaitves on the Board in order to convert this hospital into a scientific workshop and laboratory in which mere knowledge would be sought with a callous disregard of the sorrows and sufferings, and needs and lives of the poor was monstrous. Mr. Rawlings did not suggest that anything of the kind had occurred, but he was apprehensive it might have, and, therefore, medical representatives were to be excluded from the Board. He supposed it was possible at some future time that some lay officer might misappropriate the funds of the institution; therefore, men of business must be excluded from the management! ("Time.") Mr. Rawlings's humane apprehensions constituted the very strongest reason that had yet been advanced for the immediate election of medical representatives to the Board, for it was absolutely impossible for the Board as at present constituted to judge as to the propriety of any kind of treatment or procedure that might be called into question. In further remarks the speaker said he could mention numerous instances of the incompetence of the Board to deal with medical matters that came before it, but he would only quote one in which they had gone astray for want of medical assistance. Some time ago it was decided that a small charge should be made to out-patients for medicine—such charge as, in the opinion of the officials, they were able to pay. That he looked upon as an objectionable arrangement. It was a misapplication of the funds of the institution, which was not intended for patients who could pay, besides being unfair to poor chemists and druggists, whose bread was thus taken out of their mouths. By adopting this system and selling medicines the hospital was brought within the provisions of the Pharmacy Act. A large number of the medicines contained arsenic, strychnine, and other drugs which were scheduled as poisons under the Act, and the statutory regulation of the Act was that the ingredients of the medicine, together with the name of the person to whom it was sold or delivered should be entered in a book kept for the purpose, and the name and address of the seller should be affixed to the medicine. He asked whether that regulation had been complied with in this hospital? He heard on excellent authority that it had not, and therefore the Board of Management had made themselves responsible for a long series of breaches of the Pharmacy Act. Could there be anything more conclusive as to the unsoundness of the present system than the fact that the Board of Management of this great hospital were liable to be summoned for breaches of the Pharmacy Act?

Mr. Walford asked if the circular sent out by the Board of the hospital had been sent to the Governors, whose names appeared under initials in the list of governors. If so, the other people in a dispute of this sort ought to have the same privilege.

Mr. Bower said he was in a position to answer that question. Some time ago the medical staff was informed that although it was impossible to give them the addresses of governors who desired to remain anonymous, any communication which the staff wished to address to those governors would be duly forwarded. In June, 1900, when the staff issued their statement to the governors, all the copies which were intended for governors who were represented in the list by initials were forwarded by the Board to their respective destinations in order that no charge might be made that the

Board were parties to suppressing any allegations which might be made against them. (Hear, hear.) That arrangement was still in force. The Board had declined to avail themselves of any means of communication which was not equally, by their voluntary act, open to the staff. He had been anxious to intervene in this discussion for some minutes. He had the privilege not only of being a governor of the hospital, but also of having acted as solicitor, assisting the Board in the conduct of this controversy. He wished to explain in one word—and he would justify his explanation in a manner beyond doubt before he sat down—that the resolutions proposed by the Board were not designed to limit the enquiry, but rather to lead to a full and absolute enquiry into all matters, including the important one of the representation of the staff. (Hear hear.) He desired to explain how the first resolution came to be in its existing form. In January last the Board passed resolutions providing for the most ample and complete enquiry. Those resolutions were communicated by him to the medical staff, and as to the greater portion of those resolutions, they were accepted by the staff, so that the position of matters was that they had come to an agreement with the staff as to the following basis of reference: that the enquiry was to include al the charges made by their staff in the statement, the question of facilitating intercommunication between the staff and the Board, the question of the past administration of the hospital, the position, functions, and acts of the secretary-director, the existing rules and bylaws so far as any member of the commission might deem necessary, the inspection of all official books, documents, and letters which might be desired by any member of the commission, and the demand of the medical staff for electing direct representatives on the Board of Management. They were then, unfortunately, confronted by two difficulties. One was an intimation from the staff that they would resign if they did not get all they demanded. The next was that the Board unfortunately differed from the staff as to the exact form of tribunal to which these matters should be referred, and the mode in which the enquiry was to be conducted. After careful consideration, he advised the Board that it would not be preferable to put before a meeting of the governors the full resolutions, providing in detail for everything to be done. They had agreed with the staff up to a certain point, and the resolutions moved on behalf of the Board to-day simply dealt with the disputed points, and they were designed to lead to a full and absolute enquiry. ("No.") The time had now come when he was about to justify what he had said. He was authorised by the mover and seconder of the Board's resolution, and by the Board to state—and he had so intimated to Mr. Melville Green—that the Board were prepared to accept that gentleman's amendment. (Loud applause.)

Mr. M. Green said he had been asked by Mr. Bower to insert the word "including" after the figures "1900" and before the words "the facility" in his amendment. This was in order to negative any possible suggestion that the enquiry was limited in any way.

The amendment as altered was then put and unanimously agreed to.

Mr. Green said it was now necessary to decide how the enquiry should be instituted. He thought they should get together a body of enquirers who would command the confidence of everybody, and if any objection were raised to anything, that would be a reason for adopting some plan to which nobody objected, because what they all wanted was that the enquiry should result in a report being presented in which they would all have confidence. It had been suggested, and he accordingly proposed—"That a Committee of governors and subscribers (other than members of the Board or medical staff) be deputed to make all necessary arrangements for the constitution of a committee of enquiry." The present meeting was certainly too numerous to make the necessary arrangements.

Mr. Pearman said that at the time the mover of the amendment cast so many aspersions at the Board of Management he was not aware that at a Board meeting held prior to this meeting it was unanimously agreed that they would be ready to accept the amendment. That being the case he hoped the governors there and the medical staff particularly would believe that the Board of Management had no intention or desire to burke enquiry in this matter. (Hear, hear). Nothing could be further from their wish. If there was one thing that was nearer to their hearts than another it was that there should be a thorough enquiry. The charges made by the medical staff were, he was quite sure, made upon their responsibility and with the full belief on their part that they were justified in meeting them. At the same time he could not but believe—indeed, he was strongly of opinion, having sat upon the committee of enquiry which was instituted by the Board—that these charges would on investigation be proved, upon the whole, to be very much exaggerated. (Cries of "Question.") He believed they could not be maintained in the way in which they were put.

Sir J. Paget: This is surely anticipating the enquiry, and does not refer to either the resolution or the amendment.

Mr. Pearman said he made no charges against the medical staff in regard to these allegations: he was sure they were *bonâ-fide*. Every member of the staff when he signed the statement—— (Interruption). He rose for the purpose of seconding the motion.

Mr. J. Wigan hoped all were agreed that the decision of that day's meeting was vital to the hospital. He had been a Governor of that hospital for nearly forty years. He was also a Governor of St. George's, and in all the hospitals he knew of there was medical representation on the Board. He could not help thinking that they had done wisely in coming to the decision at which they had arrived. The resolution was carried unanimously.

The meeting then proceeded to the election of the committee of Governors provided for by the resolution just adopted. Several names were suggested by various Governors, and in the course of the consideration of these the chairman urged that so far as possible the names selected should be those of gentlemen who had expressed no decided opinions already. Otherwise there would be a feeling of partisanship in the matter, and that was, of course, advisable to avoid. Some of the names suggested were, for various reasons, withdrawn, including that of the Earl of Dudley. Sir Henry Burdett, who was pressed to serve, said he would be unable to do so as he was about to leave for the South of France almost immediately.

Upon Sir J. Crichton-Browne being nominated, it was urged by Mr. Bower that there should not be a preponderance of medical men on the committee.

Dr. Buzzard pointed out that Sir J. Crichton-Browne was a vice-president of the hospital, and according to the rules of the constitution of the charity he was an *ex-officio* member of the Board. Any predilection, therefore, that he might have for the medical staff might be counteracted by his being a member of the Board of Management. (Laughter.)

Sir J. Crichton-Browne said that although he was stated to be an *ex-officio* member of the Board of Management he had never received an intimation to that effect, and it was a revelation to him that he occupied that position.

Eventually Mr. Jonathan Hutchinson, F.R.S., Mr. Bischoffsheim, Mr. Melville Green, Mr. J. K. Hitchens, Sir John Paget, Bart., Miss Agnes Keyser, and Mr. J. Wigan were elected the committee. The name of Mrs. Pearman, wife of a member of the Board, was submitted to the vote, but was not accepted.

It was further resolved:—"That the Committee of Governors be instructed as follows:—To place on such committee of enquiry at least one physician or surgeon, one eminent lawer, one man engaged in commercial or financial pursuits, one man experienced in hospital management (not a medical man), and one other person, none being members of the Board or medical staff."

Mr. GREEN next moved a resolution—"To empower the committee of enquiry to conduct the enquiry as they may think fit, authorising them to request the Board and the staff to employ counsel if the committee of enquiry think necessary, and to provide that all necessary and proper expenses of the enquiry be borne by the hospital funds." The committee, he said, would of course not call for counsel if they did not want them, knowing that the expenses would come out of the hospital fund.

Sir HENRY BURDETT said he presumed the evidence or findings of the committee would be submitted to the Governors.

Mr. GREEN: That would be for the committee of enquiry to determine.

Sir HENRY BURDETT said it was a very important point. The purport of this meeting had been to take the matter out of the hands of the Board and, the staff and place it in the hands of the Governors, and although they had appointed a committee they had not given to any committee power to act on the findings of the committee of enquiry. If the matter was to be left ultimately with the Governors, provision should be made for the printing and circulation of the evidence and the report and findings of the commission amongst the Governors, who should be called together to decide upon them.

Mr. GREEN asked whether this was not met by saying that the committee might provide that all necessary and proper expenses of the enquiry be borne by the hospital funds. If the commission thought it necessary to print anything and put it before the whole body of Governors they would do it.

A GOVERNOR: Is there not some mode of settling this matter without going to such an enormous expense?

Another GOVERNOR: Surely it goes without saying that the committee is to enquire and report to the Governors.

The CHAIRMAN: Yes, certainly. I do not think it is possible to contrive any means by which this enquiry, which we hope will be full and will make a good report, should be so arranged that the Governors should be precluded from exercising their own jurisdiction. They will, of course, have to consider the report and decide upon it.

Sir HENRY BURDETT said he would second the motion if they included in it an arrangement whereby the evidence given, and the report of the commission would be circulated amongst the Governors, and submitted to a special meeting of that body. He had had a number of letters and other communications addressed to him on the subject, and there was a very strong feeling among the Governors that they would like to have the evidence before them in order that they might well weigh the matter before being asked to come to a vote upon it.

The CHAIRMAN submitted that it would be more consonant with the language of the resolution if that proposition was dealt with as a separate resolution. (Hear, hear.)

The resolution proposed by Mr. Green was then agreed to.

Sir HENRY BURDETT said that in order to satisfy what he knew to be the general feeling outside and inside the hospital among the Governors, he would move "That the committee be requested to arrange for the circulation of the evidence, report the findings of the committee amongst the Governors and their submission to a special meeting of the Governors to be called by them." The circulation of this report would cost some money, but after all, it would get some money, and the amount of money it would get, the amount of satisfaction it would give, and the amount of peace it would bring about would be worth any amount of expense. He had laboured as a Governor of this hospital to get peace and he did want a settlement which would not be artificial and incomplete, but would be perfect and satisfactory to everybody. He was taking upon himself a great responsibility—being one who advocated economy in hospitals—in making a proposal, which would entail a large expenditure; but he was so convinced of the importance and necessity of this step being taken, that, if it were necessary, he for one would be glad to share in the expense rather than forego what he was sure would in the end be the best means they could have to help the Governors in settling the matter permanently.

Mr. WALFORD seconded the proposition.

Mr. GREEN expressed the hope that the meeting would not carry the motion. It seemed to him that to impose upon the funds of the hospital the cost of printing a quantity of evidence would, unless it was really wanted, be a great mistake. Let the matter be left to the committee of enquiry, who would under the resolution already carried have powers to do what was proposed by Sir Henry Burdett. The committee, of course, must make a report, which they must necessarily print and circulate among the Governors, and if they liked to add to the report some or all of the evidence they could do so under the resolution already carried. But was it credible that the printing of the bulk of the evidence would be necessary? He thought the outcome of the enquiry would be that when the report was made, it would be assented to by everybody and commend itself to them all. If, however, it should be deemed necessary to go into the evidence, the Governors, when they had met to consider the report, could move that it all be printed for an adjourned meeting. But to impose that upon the hospital at such an enormous expense would be a very great pity, and it might do mischief, because there were things sometimes said that were better forgotten, even in giving evidence, and after it was all over they did not want to remember anything that had better be forgotten.

Mr. BOWER said he thought it would be a pity to vote on the resolution as it stood, because it could do many things that they all agreed with. It was, of course, necessary that the report should be circulated. They hoped the report, which would be the authority and pronouncement of the tribunal on the evidence, would bring peace. But was it advisable to lay down by resolution all that was said on both sides should be printed? Let them leave this to the committee, and unless Sir Henry Burdett was prepared to amend the resolution he would move that it be amended accordingly.

Mr. DIBDIN moved as an amendment that the word "evidence" be omitted from the resolution, and this was duly seconded.

The CHAIRMAN submitted the proposition "that the word 'evidence' stand as part of the question," but this was negatived. He then submitted the original resolution with the word "evidence" omitted, and it was agreed to unanimously.

Sir HENRY BURDETT moved a vote of thanks to the Lord Chancellor for presiding over the meeting. He was sure all present felt much indebted to his lordship, for not only was he always busy, but this was one of the busiest seasons of the year for him. Moreover, his lordship's presence had been a material contribution to the bringing together of the two sides, and commencing that forward progress for this hospital of union between the lay and medical management which would be so much to its benefit. If that was the result he hoped the Lord Chancellor would agree with him that his presence to-day, however inconvenient it might have been to himself, would have rendered a material service to the hospital, and not only to this hospital but to the whole of the hospitals in London.

Mr. Pearman seconded the vote, which was carried unanimously.

The Chairman, in reply, said that he was very glad that the meeting, which promised at one time to be a little excited, had ended so happily that they all agreed with each other. (Hear, hear.)

The proceedings then terminated.

